# Novel African American Colorectal Cancer *MSH3* Variants Associate With Major Genomic Instability

**DOI:** 10.1155/humu/5588764

**Published:** 2026-06-04

**Authors:** Mudasir Rashid, Hassan Brim, Shaolei Teng, Adebiyi Sobitan, Ruth Cruz-Cosme, Tang Qiyi, Minoru Koi, Katherine Casazza, Jennifer A. Surtees, John M. Carethers, Hassan Ashktorab

**Affiliations:** ^1^ Department of Medicine and Cancer Center, Howard University College of Medicine, Washington, DC, USA, howard.edu; ^2^ Department of Biology, Howard University, Washington, DC, USA, howard.edu; ^3^ Department of Medicine, University of California San Diego, San Diego, California, USA, ucsd.edu; ^4^ Department of Biochemistry, Jacobs School of Medicine and Biomedical Sciences, University at Buffalo, Buffalo, New York, USA, buffalo.edu; ^5^ Moores Cancer Center and Herbert Wertheim School of Public Health and Human Longevity, University of California San Diego, San Diego, California, USA, ucsd.edu

**Keywords:** African Americans, benchwork, colorectal cancer, CRISPR-Cas9, DNA mismatch repair, genetic variant, in silico, MSH2, MSH3, point mutation

## Abstract

Pathogenic variants of *MSH3* can increase mutational load within colorectal cells, which may drive initiation and progression of colorectal cancer (CRC). We identified several variants within *MSH3* among CRCs from African Americans (AA). To predict and assess the functional significance of these *MSH3* variants, we employed a combination of in silico analyses and in vitro functionality assays. Our objective was to elucidate the correlation between computational predictions and functional outcomes.

**Methods:**

A CRISPR‐Cas9 knock‐in approach was used to introduce and generate specific point mutations in Exons 21, 22, and 23 of *MSH3* in the wild‐type (WT) *MSH3* CRC cell line SW620, which was confirmed with Sanger sequencing. We employed cell proliferation, microsatellite instability assays, and whole genome sequencing to assess biological and genetic consequences. We utilized immunofluorescence, Western blot, and coimmunoprecipitation methods to assess subcellular localization and differences in heterodimer MSH2 binding between WT and variant MSH3 proteins.

**Results:**

We previously identified six novel, potentially pathogenic nonsynonymous variants (c.G1237A, c.C2759T, c.G1397A, c.G2926A, c.C3028T, and c.G3241A) within six exons (Exons 8 (E413K), 9 (S466N), 20 (S920F), 21 (E976K), 22 (H1010Y), and 23 (E1081K), respectively) of *MSH3* among AA CRCs as assessed by computational bioinformatic and molecular dynamic simulation analysis. We successfully knocked in three of the MSH3 variants (Exons 21, 22, and 23). Biological phenotypic assays revealed no observable changes in cell morphology or proliferation between WT and *MSH3*‐variant knocked‐in cells, and no differences were observed in microsatellite assays. Subcellular localization of variant MSH3 protein was unaffected compared with WT, whereas the interaction between MSH3 and MSH2 was not impacted. Short tandem repeats (STRs), or microsatellites, are short DNA motifs of two to six base pairs repeated consecutively, and they serve as powerful genetic markers for studying inheritance patterns and disease‐associated repeat instability. In our analysis, STR profiling revealed both repeat expansions and contractions across multiple motifs, with tetranucleotide repeats showing the most pronounced alterations. Notably, loci flanking *LINC00550* (ATTT), *FBXL7* (AGAT), and *DUSP28* (CTTT/GTTT) displayed consistent repeat expansions (+1.5 to +2), whereas intronic regions within *GPC5*, *TMEM232*, and *ABCA13* exhibited contractions. Trinucleotide STRs revealed a mix of instability, with repeat gains at *SLC25A12* and losses near *GTF3C3* and *AKAP12*. Pentanucleotide and hexanucleotide motifs were more stable overall, but expansions were still noted at *CNTN5*, *CPEB1*, and *CBSL*, and contractions at *SGO1* and *FER*. These findings highlight the bidirectional and motif‐specific nature of STR instability driven by *MSH3* deficiency. Our results support the utility of STR‐based assays as sensitive tools to detect nonclassical MSI events and deepen our understanding of *MSH3*′s role in preserving microsatellite integrity across the genome.

**Conclusion:**

This study investigated the functional consequences of MSH3 variants identified among AA CRCs. While CRISPR‐Cas9 knock‐in of MSH3 variants (Exons 21, 22, and 23) in SW620 cells did not alter cell morphology, proliferation, protein localization, or MSH2 binding, we observed genetic changes that collectively underscore the bidirectional nature of STR instability in *MSH3*‐deficient cells and reinforce this protein′s pivotal role in suppressing slippage at longer repeat motifs. This study advances our understanding of how *MSH3* deficiency contributes to genomic instability beyond canonically defined MSI loci, offering novel insights into the mutational landscapes of MMR‐deficient tumors and how these *MSH3* mutations can potentially contribute to the outcome of AA CRC patients.

## 1. Introduction

Preclinical or clinical‐based experiments conducted on biological samples, animal models, or human subjects, respectively, provide the empirical data that can be analyzed through bioinformatics pipelines [1–3]. Bioinformatic (in silico) analysis deals with collecting empirical data and using multiple computational tools, databases, and algorithms to analyze and interpret data in a fast and meaningful manner [4–6]. Nonetheless, in silico approaches present many challenges. While incorporating more biological cohort data from genomics, proteomics, metabolomics, and other omics fields that can provide a more holistic view of the biological system [7–12], in silico models typically lack the complexity of biological systems and environmental influences. Further, while using diverse experimental datasets and incorporating cellular networks, feedback loops and stochastic events enhance these models [13–23], the quality of in silico data prediction depends heavily on the quality and quantity of data used to build models. In our prior study, we conducted targeted exome sequencing on 54 African American colorectal cancers (AA‐CRCs), revealing a plethora of novel variants across different genes [24]. We were specifically intrigued by six previously unknown *MSH3* variants in AA‐CRC samples that were predicted to be deleterious [25]. Microsatellites, also known as short tandem repeats (STRs), constitute approximately 3% of the human genome. These repetitive DNA sequences, typically composed of one to six base pair motifs, are highly polymorphic and prone to length variation due to strand slippage during DNA replication. This inherent instability can contribute to genomic alterations, particularly when the cellular machinery responsible for correcting replication errors is compromised. A key component of this surveillance system is the mismatch repair (MMR) pathway, a highly‐conserved mechanism that detects and repairs DNA replication errors to maintain genomic integrity [26–28]. Central to MMR are two heterodimeric complexes: MSH2–MSH6 (MutS*α*) and MSH2–MSH3 (MutS*β*). While MutS*α* primarily recognizes base–base mismatches and small insertion/deletion loops (IDLs) of one to two nucleotides, MutS*β* is specialized in recognizing larger IDLs, particularly those involving trinucleotide, tetranucleotide, and longer nucleotide repeats. Upon recognition, these complexes initiate repair cascades to correct the mismatches and prevent mutagenesis. Deficiencies in MMR, including mutations in *MSH2*, *MSH6*, or *MSH3*, result in the accumulation of replication errors, contributing to microsatellite instability (MSI) and increased mutation rates. Mutations or reduced expression of MSH3 disrupt the stability of STRs across the genome. The resulting pattern of repeat expansions or deletions depends on the specific MSH3 alteration and the surrounding genetic context, shifting the balance of STR dynamics and shaping global mutation profiles as well as disease risk, including cancer and repeat‐expansion disorders [29–33]. Clinically, biallelic loss‐of‐function MSH3 variants cause a recessive form of colorectal adenomatous polyposis characterized by EMAST, with instability concentrated at dinucleotide and tetranucleotide repeats [34]. Experimental models (in yeast) support this mechanism that MSH3 defects preferentially produce repeat deletions, underscoring substrate specificity that differs from other mismatch‐repair proteins [30, 35]. Emerging evidence further showed that MSH3‐linked signatures can serve as biomarkers of high microsatellite instability (MSI‐H), including in subsets of tumors otherwise classified as MMR‐proficient [32, 36]. Together, these data position MSH3 as a central regulator of STR homeostasis: its disruption promotes both expansions and deletions, contributes to colorectal tumorigenesis and polyposis, and intersects with pathways relevant to cancer predisposition and repeat‐expansion diseases, with phenotypic outcomes dictated by the particular MSH3 lesion and the broader genetic background. Here we aimed to validate biological consequences of three novel *MSH3* variants (E21, E22, and E23) identified from AA CRCs with a variety of assays.

## 2. Material and Methods

### 2.1. Cell Lines and Reagents

The colorectal cancer cell line (SW620) was cultured in RMPI medium (RPMI 1640 Medium, #61870036) with 10% FBS (fetal bovine serum, qualified, heat inactivated, United States #16140071) and 1% pen–strep antibiotic for 5% CO_2_ in a 37°C incubator. The materials included synthetic donor DNA oligos, gRNA, Cas9 protein (# PECAS9–PURedit, Sigma) transfecting reagent lipofectamine LTX Reagent PLUS Reagent (Thermo; #15338030), Opti‐MEM I Reduced Serum Medium (#31985062,Thermos), Genomic DNA kit (#K0722), GeneJET Plasmid Miniprep Kit (#K0503), *MSH3* specific exon primers and specific donor DNA for *MSH3* CRISPR‐Cas9 (mentioned in Tables S1 and S2), DreamTaq Green PCR Master Mix (2X× #K1081), TrackIt 100 bp DNA Ladder (#10488058); Magnetic IP/Co‐IP Kit (Pierce Classic #88804); TA cloning pGEM‐T Vector Systems (#A3600,Promega). Antibodies anti‐MSH3 (#22393‐1‐AP), anti‐MSH2 (#33‐7900, Invitrogen), Anti‐H3 (Histone H3 Polyclonal Antibody #PA516183), anti‐beta tubulin (4E4, Catalog #MA5‐47432) were used for different experiments; HRP‐conjugated antimouse (Goat antimouse IgG (H + L) eecondary antibody, HRP #31430) and antirabbit (Goat antirabbit IgG [H + L] secondary antibody, HRP, #31460) antibodies were purchased from Invitrogen. Alexa 488 conjugated antimouse antibody and Alexa 589 conjugated antirabbit antibody were from Invitrogen, MTT (3‐(4,5‐Dimethylthiazol‐2‐yl)‐2,5‐diphenyltetrazolium bromide) (#M6494); DAPI and Hoechst Nucleic Acid Stains (#H3569); signals were detected using SuperSignal West Pico (Thermo Scientific, Waltham, Massachusetts) and captured by an iBright instrument (iBright FL1500 Imaging System).

### 2.2. Bioinformatic (In Silico) Analysis

Our previous bioinformatic (in silico) data showed that six *MSH3* nonsynonymous variants are novel and predicted to be deleterious in nature based on multiple bioinformatic analyses such as molecular dynamic simulation and other tools/databases and algorithms. The variants c.G1237A, c.C2759T, c.G1397A, c.G2926A, c.C3028T, and c.G3241A corresponded to MSH3 amino‐acid changes p.E413K, p.S466N, p.S920F, p.E976K, and p.H1010Y, p.E1081K in six exons (8, 9, 20–23) and of *MSH3*, respectively [25]. To verify our previous in silico findings, therefore, we used another *bioinformatics* structure‐based computational tool—FoldX [37], a computational tool used to analyze the impact of atomic and molecular energies within a protein structure focusing on Gibb′s free energy (*Δ*G). FoldX evaluates how individual energies contribute to the overall *Δ*G of the protein, particularly when missense variants occur. Missense variants can alter the *Δ*G, affecting the protein′s stability. FoldX predicts changes in *Δ*G (*ΔΔ*G) due to variants, with deviations of about 0.5 kcal/mol from experimental values. Variant changes above +0.5 kcal/mol are considered destabilizing (pathogenic), whereas those below −0.5 kcal/mol are stabilizing

ΔΔGStability=ΔGMutant−ΔGWild−type

Additionally, FoldX predicts the impact of missense mutations on protein–protein interactions. Variants increasing interaction energies weaken binding affinity, whereas those decreasing interaction energies strengthen it:

ΔΔΔGBinding−Affinity=ΔΔGMutant−ΔΔGWild−type

The MSH3–MSH2 (MutS*β*) complex model was constructed using AlphaFold3, an advanced tool that accurately predicts protein structures and interactions [38]. This model was used to investigate the impact of mutations on the stability and binding affinity of the MSH3–MSH2 complex. AlphaMissense [39] and Meta‐SNP [40] were used to predict the pathogenicity of the variants, providing insights into their potential disease‐causing effects.

### 2.3. CRISPR‐Cas9 Gene Editing and Transfection

SW620 cells were plated onto 24‐well plates at 1 × 10^6^ cells per well in 500 *μ*L of growth medium followed by 30%–70% confluence. Before transfection, cells were serum starved for 18 h followed by 8 h of release generating late S phase or G2 phase cell cycle arrest, because homology‐directed repair (HDR) pathway is more proficient in this phase of the cell cycle [41]. In brief, cells were electroporated with an RNP complex and donor DNA using the Gene Pulser Xcell Total System (#1652660) and a transfection reagent. Synthetic single guide RNA for each MSH3 mutant (Exons 21, 22, and 23) was prepared at 100 *μ*M in a 10‐mM Tris buffer, pH 7.4, alongside donor DNA. PURedit Cas9 was reconstituted in 50 *μ*L of solution (approx. 5 mg/mL), mixed gently, incubated on ice for 30 min, and stored at −20°C for frequent use or −80°C for long‐term storage. The RNP complex was prepared at a 5:1 molar ratio of guide RNA to Cas9 protein, with optimization as needed. On transfection day, 25 *μ*L of Opti‐MEM medium, 500 ng Cas9 nuclease (~5 *μ*g/mg), and 125 ng gRNA were combined and incubated at 25°C for 5–10 min to form RNPs. Donor DNA (500 ng single‐strand) was added after 10 min. Separately, 25 *μ*L of Opti‐MEM medium and 1.5 *μ*L of Lipofectamine were mixed, incubated at 25°C for 5 min, and combined with the RNP complex. This final mixture was incubated for 10–15 min to form Cas9 RNP–lipofectamine complexes before being added to cells. Sequence details are available in Tables S1 and S2.

### 2.4. Screening and Validation of CRISPR‐CAS Knock‐in Mutation by Sanger Sequencing

After 48–72 h of posttransfection, the cells were serially diluted in 24‐well plates and cultured again for 72 h followed by harvesting and determination of successful genome editing by isolating genomic DNA (Catalog #K0722, Thermo) from each 24‐well clone as per the manufacturer′s protocol. The quality and quantity were assessed followed by amplification of *MSH3* Exons 21, 22, and 23 using specific PCR primers, as given in Table S1. Each of the amplified products was processed for TA cloning, and each bacterial colony was screened by isolating plasmid from each of the 24 clones and validated by Sanger sequencing. Once positive clones of each respective variant (SW620 with Exons 21, 22, and 23 positive clones) were obtained, they were cultured constantly until Passage 7 to be utilized for functional assays.

### 2.5. Functional Assays for Validated Variant (*MSH3* Exons 21, 22, and 23) SW620 Cells

Wild type (WT) and variant (Exons 21, 22, and 23) Passage 7 SW620 cells were seeded followed by 70% confluent cells that were subjected to various assays such as proliferation, apoptotic assay, Western blot, immunofluorescence (IF), coimmunoprecipitation and MSI/EMAST assays.

#### 2.5.1. MTT Cell Viability Assay

Short‐term proliferation assay was performed as per the previous study [42] in brief, WT and variant Passage 7 SW620 cells (3 × 10^6^) were seeded in a 96‐well plate and incubated for 24 h. A 5‐mg/mL MTT stock solution was prepared, sterilized, and 10 *μ*L was added to each well. After 2–5 h of incubation at 37°C, 100 *μ*L of DMSO was added to each well and incubated for 15 min. Absorbance at 570 nm was then measured. The assay detects viable cells by the reduction of MTT to a purple formazan product, quantified spectrophotometrically.

#### 2.5.2. Clonogenic Assay

Long‐term survival assay of WT and variant Passage 7 SW620 cells (1 × 10^3^) were seeded in 60 mm^2^ culture dishes. Media was changed at regular time intervals, and colonies were allowed to form for 14 days. Postincubation, colonies were fixed with 4% paraformaldehyde for 30 min at room temperature, washed with 1× PBS, and stained with 0.5% crystal violet in 50% methanol for 1 h. The excess stain was removed by washing the plates in water, and colonies were counted under a light microscope, and a bar graph was plotted. Statistical significance was determined by conducting an unpaired student′s *t*‐test.

#### 2.5.3. Apoptotic Assay

WT and variant Passage 7 SW620 cells (3 × 10^6^) were seeded in 96‐well microplates (black wells/clear flat bottom) and incubated for 24 h. After incubation, cells were washed one to two times with 100 *μ*L assay buffer by carefully pipetting up and down, followed by adding 200 *μ*L of assay buffer and 2 *μ*L of Apopxin Green Indicator (100×; Green [Ex/Em = 490/525 nm] for apoptotic cells), 1 *μ*L of 7‐AAD (200×; Red [Ex/Em = 550/650 nm] for late apoptotic and narcotic cells), and 1 *μ*L CytoCalcein 450 (200×; Blue for viable cells) to cells. Cells were incubated at room temperature for 30–60 min, followed by 2× washing with 100–200 *μ*L assay buffer. Finally, 100–200 *μ*L assay buffer were replaced and cells were analyzing under the fluorescence EVOS microscope, Additionally, SW620 WT cells were induced by 1 *μ*M staurosporine (#Catalog No. T6680) an apoptotic agent for 3 h used as a positive control for the experiment.

#### 2.5.4. Subcellular Fractionation, Western Blot, and IF Analysis

WT and variant Passage 7 SW620 cells were seeded onto 90‐mm plates and at 80% confluency cells were harvested and lysed with lysis buffer, and nuclear/cytoplasmic separation was conducted as per the manufacturer′s protocol. (Cat #78835). Protein concentration was determined using the BCA protein assay kit (#23227). Protein lysates and nuclear and cytosolic fractions were separated by 4%–20% Tris‐Acetate SDS‐PAGE (Invitrogen) and transferred onto nitrocellulose membrane as descried previously [43]. Immunodetection was done using primary antibodies such as antihuman MSH3 rabbit monoclonal antibody, antihuman MSH2 mouse monoclonal antibody, antihuman H3 rabbit monoclonal antibody, and anti–b‐tubulin antibody for the detection of specific proteins, followed by use of horseradish peroxidase‐conjugated secondary antibody such as Goat antimouse antibody and Goat antirabbit antibody. Signals were detected and captured using by an iBright FL1500 Imaging System, and data were analyzed by iBright analysis software.

#### 2.5.5. Immunocytochemistry

IF was performed as described previously [44, 45]. In brief, WT and variant Passage 7 SW620 cells were seeded on cover slips, incubated overnight, and fixed with 4% paraformaldehyde. Cells were permeabilized with 0.5% Triton X‐100, blocked with 3% BSA, and incubated overnight with the primary antibody (MSH3, 1:100) at 4°C. After washing, cells were incubated with a fluorophore‐conjugated secondary antibody, stained with DAPI, and mounted. Images were captured and analyzed using the EVOS M7000 microscope.

#### 2.5.6. Coimmunoprecipitation

WT and variant cells Passage 7 SW620 cells were seeded onto 90‐mm plates, and 80% confluent cells were harvested and lysed into lysis buffer as per the manufacturer′s protocol (Kit; #88804). In brief, culture medium was removed from confluent cells and washed with cold 1× PBS. After that, 500 *μ*l ice‐cold IP Lysis/Wash Buffer was added and incubated for 5 min with periodic mixing, followed by centrifugation at ~13,000 × g for 10 min to pellet the cell debris, and supernatants were transferred to fresh tubes for protein concentration determination by BSA (Pierce, BCA Protein Assay) and further analysis. A total of 2–10 *μ*g of affinity‐purified antibody was added to 2–10 *μ*g cell lysate, and the antibody/lysate solution was diluted further with 500 *μ*L of IP Lysis/Wash Buffer and incubated for 1–2 h at room temperature or overnight at 4°C to form the immune complex as previously described [46].

#### 2.5.7. MSI/EMAST Assay

WT and variant cells Passage 7 were seeded onto 90‐mm plates, and 80% confluent cells were harvested and subjected to DNA isolation as per the manufacturer′s protocol. MSI/EMAST were performed as previously described [43]. In brief, DNA was extracted and subjected to multiplex polymerase chain reaction (QIAGEN, Hilden, Germany) to determine MSI and EMAST presence/absence of combined 14 microsatellite markers (two mononucleotide [*BAT25* and *BAT26*], five dinucleotide [*D2S123*, *D5S346*, *D17S250*, *D18S64,* and *D18S69*], and seven tetranucleotide microsatellite sequences [*D9S242*, *D20S82*, *D20S85*, *D19S394*, *D8S321*, *MYCL1*, and *RBM47*]) as previously described [47–49] in four reactions simultaneously with different annealing conditions. Cycling conditions for *D2S123*, *D5S346*, *D17S250*, and *MYCL1* were 52°C for 30 s. All the primer sequences for MSI and EMAST markers are listed in the Table S3.

#### 2.5.8. Whole‐Genome Sequencing (WGS) of SW620‐WT and CRISPR Knock‐in Clones (SW620‐E21, E22, E23)

Genomic DNA was extracted from parental SW620 WT cells and the three CRISPR‐Cas9 knock‐in clones (SW620‐E21, SW620‐E22, and SW620‐E23) using a Kit (ThermoScientific, #K0722) according to the manufacturer′s protocol. DNA quality was assessed via agarose gel electrophoresis and quantified using both NanoDrop spectrophotometry and the Qubit dsDNA BR Assay Kit to ensure sufficient purity and concentration for library preparation. WGS was performed by Novogene, and libraries were prepared using the Illumina TruSeq DNA PCR‐Free Library Prep Kit. Briefly, 50 ng of high‐quality genomic DNA from each sample was fragmented to ~350 bp using an ultrasonicator, followed by end‐repair, A‐tailing, and ligation of indexed Illumina adapters. The libraries were validated using an Agilent 2100 Bioanalyzer and quantified by qPCR. Sequencing was performed on an Illumina NovaSeq 6000 system, generating 150 bp paired‐end reads, with an average genome‐wide coverage target of ~30× per sample. Raw sequencing reads were assessed for quality using FastQC and trimmed with Trimmomatic to remove low‐quality bases and adapter sequences. Cleaned reads were aligned to the human reference genome (GRCh38/hg38) using BWA‐MEM, followed by duplicate marking with Picard and base quality score recalibration using GATK. Mapping statistics, depth of coverage, and genome completeness were evaluated with SAMtools and Qualimap to ensure uniform coverage across all samples. The resulting BAM files were used for variant calling, structural variant detection, and downstream analyses including off‐target mutation screening and genomic integrity comparisons among WT and mutant clones.

##### 2.5.8.1. Off‐Target Analysis of CRISPR‐Cas9 Edited Clones

Sequencing reads from WGS were aligned to the human reference genome (hg38) using BWA‐MEM, and Variant calling was performed using GATK HaplotypeCaller, and resulting VCF files were filtered for high‐confidence single nucleotide variants (SNVs) and small insertions/deletions (INDELs), and STR between WT and variant clones. Variants were annotated using ANNOVAR and filtered against a list of predicted off‐target sites that were analyzed by in silico tools using CAS‐OFFINDER (http://www.rgenome.net/cas-offinder/) with “PAM type” of SpCas9 from *Streptococcus pyogenes*: 5 ^′^‐NGG‐3 ^″^, maximum number of mismatches 3, bulge sizes of 0 against the “Homo sapiens (GRCh38/hg38)—Human” genome. We used both the cRNA sequence and the complementary sequences. The predicted off‐target locations were then matched to the actual locations of the variants in each sample. We did not find a significant number of variants that overlapped the predicted off‐target mutant MSH3 (E21, E22 and E23) compared with WT MSH3 SW620 parental cells.

##### 2.5.8.2. STR Profiling

STR was done using the HipSTR software suite (https://hipstr-tool.github.io/HipSTR/). We used a predefined set of known human hg38 STR locations downloaded from the HipSTR References site (https://github.com/HipSTR-Tool/HipSTR-references). To that list, we added two additional sites (CNBP STr site at chr3:129172577‐129172656 and MYCL EMAST STR site at chr1:39917235‐39917414). The HipSTR analysis was done using parameters max − str − len = 250 and min − reads = 5 using all the samples together. The detected STRs were annotated using Annovar (http://annovar.openbioinformatics.org/en/latest/) and sorted by the difference in the number of repeats in control versus treated.

##### 2.5.8.3. Visualization

Circos plots were generated using Circos software (Version 0.69.9) to visualize genomic differences. Each plot includes: Track 1—Outermost black ring with human chromosomes (1–22, X) and cytoband labels; red lines within cytobands denote centromere positions. Track 2—The second following ring is composed of red (positive difference in number of repeats between treated and WT) and blue (negative difference in number of repeats between treated and WT) dots which show the STRs with five or more nucleotide lengths. Track 3—The third following ring with green (positive) and purple (negative) points are the tetranucleotide (four nucleotide) STRs. Track 4—The fourth following ring with orange (positive) and yellow (negative) are the three or less nucleotide STRs. Tracks 5 and 6—The fifth light red ring is the number of SNVs in 10 Mb, and the innermost blue ring is the number of INDELs in 10 Mb. Each experimental sample was compared directly with the WT to highlight differences in the aforementioned genomic features.

## 3. Results

In our prior study, we conducted targeted exome sequencing coupled with multiple bioinformatic pipelines and analysis (in silico) to identify various genetic variants in a number of genes among AA‐CRC samples. Here, we present additional computational analyses of six *MSH3* variants (c.G1237A, c.G1397A, c.C2759T, c.G2926A, c.C3028T, and c.G3241A) that corresponded to MSH3 amino‐acid changes (p.E413K; p.S466N; p.S920F; p.E976K; p.H1010Y; and p.E1081K) in six exons (8, 9, and 20–23), respectively. E8 and E9 are in MSH3′s Domain II, which facilitates communication between the DNA‐binding and ATPase domains of the complex. E20–23 are in MSH3′s Domain V, which encodes the dimerization and ATPase domains of the complex. Our previous report suggested novelty and their potential pathogenic nature of these six MSH3 variants in AA‐CRCs [25].

### 3.1. Effects of MSH3 Point Mutations on Protein Function Using Bioinformatics Approaches

**Figure 1:**
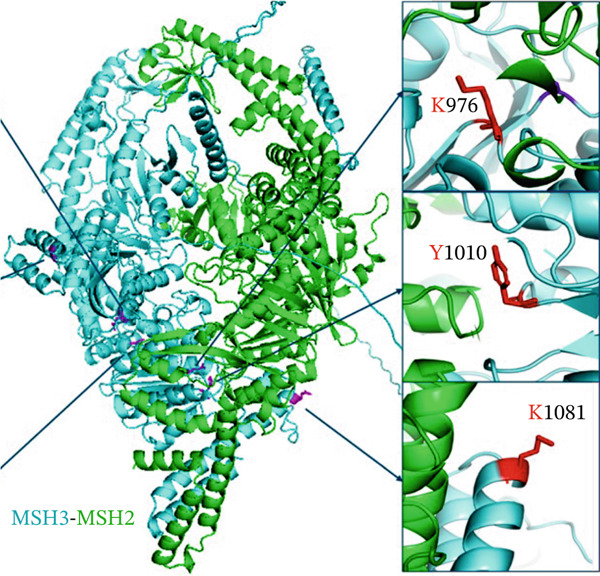
Structural representation of key MSH3 point mutations. The locations of wild‐type residues (magentas) are marked in the complex of MSH3 (cyan) and MSH2 (green). The variant residues (red) are shown in the subfigures.

We applied the structure‐based computational tool, FoldX, to analyze further the potential effects of these patient‐derived point mutations on MSH3 protein stability and MSH3–MSH2 interaction. The computational analysis using FoldX indicated that residues E976K (E21) and H1010Y (E22) are located at or near the interface with MSH2 (Figure 1). Notably, E976 (E21) is part of the conserved Walker B motif that binds Mg^2+^ and is required for ATP hydrolysis. Computational predictions indicate that the E976K (*Δ* *Δ*G = 0.21 kcal/mol) and H1010Y (*Δ* *Δ*G = 0.27 kcal/mol) variants have negligible effects on the structural stability of MSH3 (Table 1) and do not lead to steric hindrance. However, interaction analysis predicted that the E976K variant (*Δ* *Δ*G = 0.789 kcal/mol) weakens the MSH3–MSH2 complex, whereas the H1010Y variant (*Δ* *Δ*G = −0.606 kcal/mol) is predicted to enhance the MSH3‐MSH2 binding interface. FoldX predicted the E1081K (E23) variant suggest destabilizing the MSH3 structure (*Δ* *Δ*G = 1.28 kcal/mol) while maintaining an unaffected binding affinity between MSH3 and MSH2 (*Δ* *Δ* *Δ*G = −0.196 kcal/mol).

**Table 1:** Bioinformatic predicted effects of MSH3 variants on protein stability, binding affinity, and pathogenicity.

MSH3 exons (mutation)	Structure‐based analysis				Sequence‐based analysis			
	FoldX				Alpha‐missense		Meta‐SNP	
	*Protein stability* (*MSH3*)		*Binding affinity* (*MSH3-MSH2*)		*Mutation pathogenicity*			
	*Δ* *Δ* *G*	*Effect*	*Δ* *Δ* *Δ* *G*	*Effect*	*Score*	*Effect*	*Score*	*Effect*
E21 (E976K)	0.21	Neutral	0.789	Weakens	0.992	Likely_pathogenic	0.775	Disease
E22 (H1010Y)	0.27	Neutral	−0.606	Strengthens	0.982	Likely_pathogenic	0.784	Disease
E23 (E1081K)	1.28	Destabilizing	−0.196	Neutral	0.1	Likely_benign	0.155	Neutral

The sequence‐based analysis using AlphaMissense and Meta‐SNP tools suggests that the Exons E976K (E21) and H1010Y (E22) variants are likely pathogenic and disease‐causing, respectively (Table 1). In contrast, E1081K (E23) is predicted as a likely benign and neutral variant, as shown in Table 1 and Figure 1. These analyses predicted that E20–E23 variants would each have some functional consequences. Therefore, we focused the p.E976K (E21), p.H1010Y (E22), and p.E1081K (E23) of the study on these mutations.

### 3.2. Knock‐in Point Variants Using CRISPR‐Cas9‐HDR in SW620 Cells and Validation

**Figure 2:**
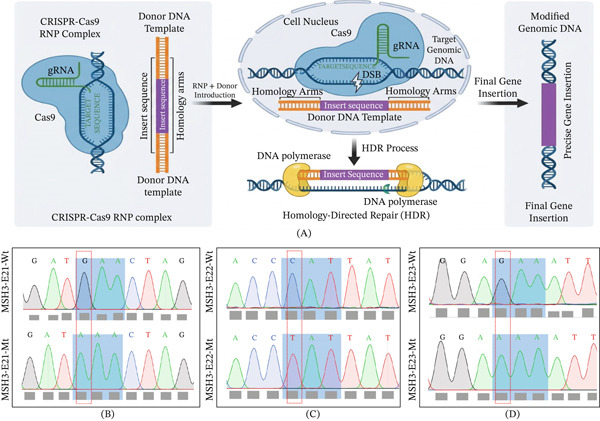
Schematic representation of step‐wise MSH3 gene editing via direct delivery of CRISPR/Cas9‐RNP complex and validation by Sanger sequencing: (A) Formation of CRISPR/Cas9‐RNP complex, transfection of RNP complex, target cleavage and double strand DNA (DBS) formation, enhance HDR mechanism in cells repair and repair double‐strand DNA breaks induced by Cas9 enzyme, screening of point mutation using via isolation of gDNA, gene specific PCR and followed by sanger sequencing; homology‐directed repair (HDR) single guide RNA (sgRNA), clustered regularly interspaced short palindromic repeats, and CRISPR‐associated protein 9 (CRISPR/Cas9), RNP (ribonucleoprotein). (B–D) The representation of variants (E21, E22, and 23) of with wild (Wt) and variant type (Mt) chromatogram; gray shaded with red marked (dotted box) represents the (B) chromatographs for DNA sequencing of Exon 21 substitution in Codon 2926 (GAA to AAA); (C) Exon 22 substitution in Codon 3028 (CAT to TAT); and (D) Exon 23 substitution in Codon 3241 (GAA to AAA) of MSH3 validating knock‐in point variants by Sanger sequencing.

Based on in silico data, we delved deeper into the biological significance of the Domain V *MSH3* variants and explored any potential associations they may have with AA‐CRC development and progression. We introduced the point variants into the WT MSH3 cell line SW620 using a CRISPR‐Cas9–HDR–based approach. We successfully introduced and generated three *MSH3* variants: (a) Exon 21 substitution in Codon 2926 (GAA to AAA), (b) Exon 22 substitution in Codon 3028 (CAT to TAT), and (c) Exon 23 substitution in Codon 3241 (GAA to AAA), with the modifications meticulously confirmed by isolation of gDNA from SW620 cells, then TA cloning followed by validation using Sanger sequencing (Figure 2A–D). This precise genetic manipulation enabled us to ultimately utilize the variant cells to examine the functional consequences of variant *MSH3*.

In CRISPR‐editing mutation‐function experiments, it is always required to do an off‐target analysis to rule out functional changes due to secondary/unwanted targets editing. This analysis led to no off‐target mutations being detected in E21 and E23 knock‐in point mutations compared with parental cells. For E22 knock‐in, the in silico analysis revealed one likely off‐target at chr1_434237 that was not present in the sequence edited cells after bioinformatics analysis.

### 3.3. Functional Assessment of Three CRISPR‐Cas9 Knock‐in Point Variants

**Figure 3:**
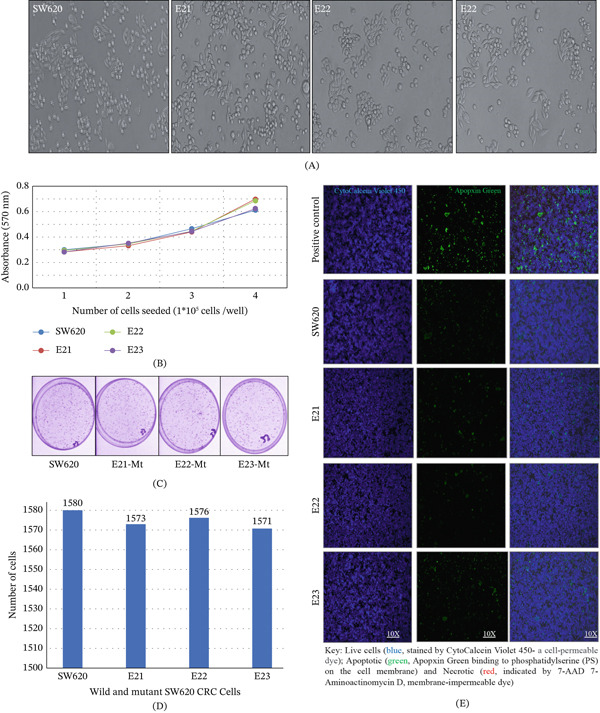
Phenotypic impact of MSH3 variants in SW620 cells: (A) Cell morphology was assessed using upright microscope; (B–D) short‐ and long‐term proliferation assay was performed using MTT and clonogenic assay; MTT (3‐(4,5‐dimethylthiazol‐2‐yl)‐2,5‐diphenyltetrazolium bromide); (E) apoptotic assay was performed using wild and variant SW620 MSH3 cells; Apopxin Green Indicator (100×; green [Ex/Em = 490/525 nm] for apoptotic cells) and 7‐AAD (200X; Red [Ex/Em = 550/650 nm] for late apoptotic and narcotic cells) and CytoCalcein 450 (200×; blue for viable cells); and SW620 wild‐type cells were induced with staurosporine, an apoptotic agent used as a positive control for the apoptotic experiment.

Functional validation of CRISPR‐Cas9 knock‐in point variants of *MSH3* Exons 21, 22, and 23 was explored to further investigate their association with AA‐CRC pathogenesis. Variant calling was followed by annotation using ANNOVAR, and potential off‐target sites were filtered based on predictions generated by CRISPOR and Cas‐OFFinder, using the corresponding guide RNA sequences (Table S2). To confirm the absence of off‐target effects, predicted sites were manually inspected using Integrative Genomics Viewer (IGV). Comparative analysis between WT and edited clones revealed no evidence of unintended mutations in or near coding regions or regulatory elements, supporting the high specificity of the gRNA designs used in this study. Upon validating genome integrity, we next assessed the functional consequences of the MSH3 point mutations. A series of phenotypic assays—including morphological assessment, cell proliferation (MTT and clonogenic assays), and apoptosis analysis—were conducted using both WT and mutant SW620 cell lines. We observed no discernible changes in cell morphology (Figure 3A); long‐ and short‐term proliferation assays did not display any significant alterations from control (Figure 3B–D); apoptotic assay did not show any significant change between WT and variant *MSH3* SW620 cells (Figure 3E). These results indicate that these three point *MSH3* variants within Exons 21, 22, and 23 do not significantly affect the cellular phenotypic behavior of SW620.

### 3.4. Subcellular Localization and MSH2 Interaction of WT and Variant MSH3

**Figure 4:**
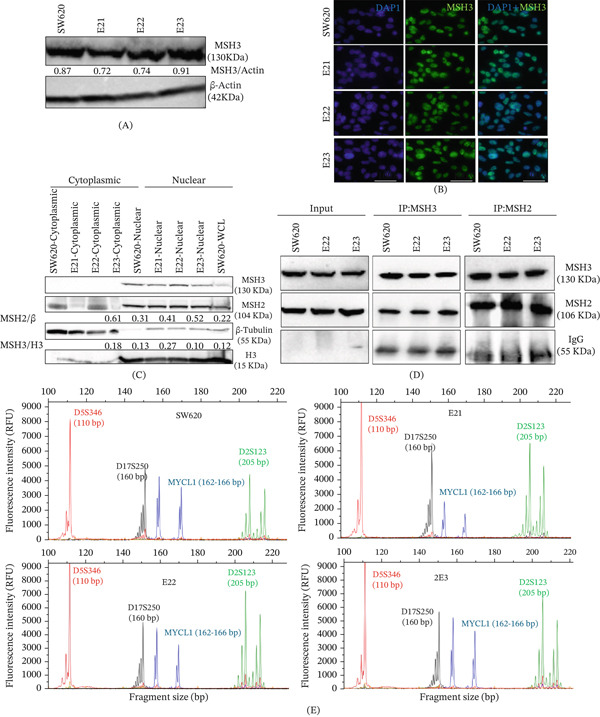
Expression, subcellular fractionation, and interaction of wild‐type and variant MSH3 evaluating genome stability: (A) Whole cell lysate of SW620 and mutant MSH3 Exons 21, 22, and 23 were prepared followed by Western blot, and expression of MSH3 was determined. (B–C) Localization of MSH3 was assessed by cytoplasmic and nuclear fractions using immunofluorescence and Western blot analysis. (D) Coimmunoprecipitation followed by Western blot analysis of wild‐type and variant Exons 21, 22, and 23 SW620 cells, respectively. (E) Microsatellite instability/elevated microsatellite alterations at selected tetranucleotide repeats (MSI/EMAST) assay for SW620, E21, E22, and E23 using fragment analysis capillary electrophoresis with specific EMAST markers blue: MYCL1 (TNR), green: D2S123 (DNR), black: D17S250 (DNR), red: D5S346 (DNR); DAPI a nuclear counterstain; H3 and beta tubulin as nuclear and cytoplasmic marker, respectively; 100× magnification; IP‐immunoprecipitation, IgG‐immunoglobulin as negative control; MNR (mono‐nucleotide repeat), DNR (di‐nucleotide repeat), TNR (tetranucleotide repeats).

We conducted further experiments to comprehensively understand the effects of WT and variant *MSH3* SW620 cells, examining expression levels, subcellular localization, and binding between MSH2 and MSH3 using immunoblot, IF, and coimmunoprecipitation assays, respectively. MSH3 expression was not altered between WT and variants (Figure 4A), and subcellular localization of MSH3 protein remained nuclear between control and variants, as cytoplasmic translocation is a common way to inactivate MSH3 (45, B) (Figure 4B–C). We found no differences in protein interaction between MSH3 and MSH2 proteins with WT and variant MSH3 (Figure 4D). MSI/EMAST assay was performed comparing WT and different variants of MSH3 in SW620 cells. We observed no differences and lack of generation of MSI or EMAST in both the control and variant MSH3 SW620 cells (Figure 4E).

### 3.5. Genomic Landscape of MSH3 Knock‐in Clones Reveals Widespread Mutational Burden

**Figure 5:**
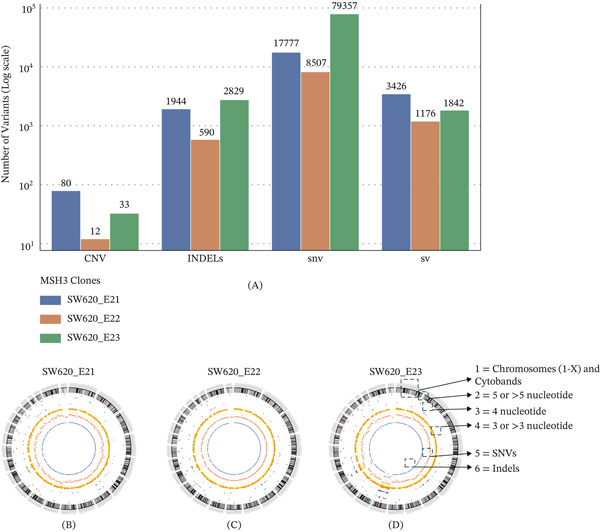
Genome‐wide impact of CRISPR‐Cas9 knock‐in mutations in MSH3 clones visualized by Circos plots and mutation burden comparison: Genome‐wide structural and sequence alterations were assessed in three CRISPR‐Cas9–edited SW620 clones (SW620_E21, SW620_E22, and SW620_E23), each harboring a point mutation in MSH3 Exons 21, 22, or 23, respectively. (A) The bar graph shows the number of copy number variations (CNVs), insertions/deletions (INDELs), single nucleotide variants (SNVs), and structural variants (SVs) for each variant type across clones SW620_E21, SW620_E22, and SW620_E23. (B–D) Circos plots visualizing genome‐wide differences in STRs, SNVs, and INDELs in (B) SW620_E21, (C) SW620_E22, and (D) SW620_E23 compared with WT SW620 cells. Each Circos plot integrates multiple tracks, offering a spatial overview of mutation distribution across chromosomes. Track 1 (Outer ring): Human chromosomes (1–22, X) labeled with cytobands; red lines mark centromere positions. Track 2: STRs with repeat units ≥ 5 nucleotides. Red dots: repeat gain; blue dots: repeat loss. Track 3: Tetranucleotide STRs (4‐nt). Green dots: repeat gain; purple dots: repeat loss. Track 4: STRs with ≤ 3 nucleotides. Orange dots: gain; yellow dots: loss. Track 5: SNV density per 10 Mb (light red). Track 6: INDEL density per 10 Mb (blue).

WGS revealed substantial differences in mutational burden across the MSH3 knock‐in clones when compared with the parental SW620 line (WT for MSH3 gene). Notably, the SW620_E23 clone exhibited the highest number of SNVs (79,357), INDELs (2829), and structural variations (SVs, 1842), whereas SW620_E21 had elevated levels of copy number variations (CNVs, 80) and SVs (3,426). In contrast, the SW620_E22 clone showed relatively fewer alterations across all categories shown in Table 2 and Figure 5A. These observations uncover the extent of genomic alterations, including STR results we observed due to CRISPR–Cas9–mediated genome engineering with targeted and technically precise knock‐in point mutations within MSH3 Exons 21, 22, and 23, with broad genomic alterations compared with parental controls. Therefore, how often such changes occur and to what degree they influence downstream biological processes remain an important consideration.

**Table 2:** Summary of genomic variant burden in MSH3 knock‐in clones.

Sample	CNV	INDEL	SNV	SV
SW620_E21	80	1944	17,777	3426
SW620_E22	12	590	8507	1176
SW620_E23	33	2829	79,357	1842

Abbreviations: CNV, copy number variants; INDEL, insertions/deletions; SNV, single nucleotide variant; SV, structural variants.

To further investigate the genome‐wide consequences of CRISPR‐Cas9–mediated knock‐in mutations targeting MSH3 Exons 21, 22, and 23, we performed STR profiling using whole‐genome variant data derived from edited SW620 clones. These profiles were directly compared with the parental SW620 WT cell line to assess differences in sequence‐level and structural genomic features. Circos plots were generated using Circos software (Version 0.69.9), providing a circular visualization of genomic alterations across all human chromosomes. The plots integrate multiple data layers, enabling spatial mapping of STR variation, SNVs, and INDELs, as shown in Figure 5B–D.

Hierarchical clustering heat maps were generated to visualize changes in STR repeat counts across di‐, tri‐, tetra‐, penta‐, and hexanucleotide motifs. Each STR locus was annotated with genomic coordinates, associated gene(s), motif type, and the minimum observed difference between wild‐type and mutant clones. In the trinucleotide STRs (Figure S1A), we observed a combination of STR instability patterns that included both expansion and contraction events in MSH3‐edited clones as compared with SW620 WT cells. Notably, loci such as *SLC25A12* (chr2, TGC motif) and *ZFP92* (chr16, AAC motif) showed consistent expansions, with repeat counts increasing by approximately +0.5 to +1.0 across all three MSH3 knock‐in clones, indicating gain‐of‐repeat events potentially due to uncorrected insertion slippage in the absence of functional MSH3 activity. These expansions may represent a shift toward MMR deficiency. Conversely, STR contraction events were observed in genes like *RAB11FIP2* (chr8, AAT), *AKAP12* (chr6, GAA), and *GTF3C3* (chr2, GTT), where repeat lengths decreased by 0.5–1.0 repeats in edited clones relative to WT. These loci likely reflect unresolved deletion‐prone mismatches that accumulate without MSH3‐mediated repair, supporting the bidirectional nature of MSI. These findings suggest that MSH3 not only prevents repeat expansions but also stabilizes fragile loci prone to contraction, particularly in genes involved in transcriptional regulation and signaling. Interestingly, a significant subset of trinucleotide loci remained unaffected by MSH3 editing, including *MYT1L* (chr2, GAA), *ZNF430* (chr19, AAT), and *SLC9A4* (chr2, TTC), where repeat counts were identical across all samples. This indicates that some genomic regions possess inherent STR stability, possibly due to favorable sequence context or chromatin configuration that reduces polymerase slippage. In the tetranucleotide STR category (Figure S1B), MSH3‐edited SW620 clones (E21, E22, and E23) exhibited both repeat expansions and contractions at multiple loci compared to the WT background, reflecting a clear pattern of bidirectional MSI. The most prominent expansion was observed at an intergenic region adjacent to *LINC00550* on chromosome 13, which harbored an ATTT repeat motif. In this locus, repeat counts increased consistently from 9 in WT to 11 in all three mutant clones, representing a significant +2.0 repeat gain. This repeat expansion was uniform across all edited samples, indicating a stable gain‐of‐function slippage event likely uncorrected due to MSH3 disruption. Other notable expansions included the AGAT‐motif locus flanking *FBXL7* and *MARCHF11* on chromosome 5, where repeat counts rose from 8 in WT to 9.5 in the edited clones, and a region within the 3 ^′^ UTR of *DUSP28* (CTTT/GTTT motif, chr2), which showed a repeat gain from 4.5 to 6.0 across knock‐in samples. These loci, enriched in A/T tetranucleotide motifs, appear to be particularly prone to polymerase slippage when MSH3‐mediated repair is compromised. In contrast, contraction events were frequently observed in intronic and intergenic loci. For example, *GPC5* (chr13, AAAT), *ABCA13* (chr7, AAAC), *TMEM232* (chr5, AAAC), and *RBM24* (chr6, AAAT) showed repeat losses ranging from −1.0 to −1.5. Such consistent contraction patterns suggest that without functional MSH3, deletion‐prone mismatches accumulate, especially at AT‐rich repeat regions, further supporting the role of MSH3 in correcting both insertions and deletions at microsatellite loci. Additionally, loci such as *LIN52*, *FRG1DP*, *PRKAR1B*, and *H2BC11* remained relatively stable across all samples, highlighting that STR susceptibility to instability may depend on genomic context, motif composition, or local chromatin environment. Together, these results underscore MSH3’s pivotal function in maintaining tetranucleotide STR stability. The pattern of both expansions and contractions, primarily at A/T‐rich loci, supports the hypothesis that MSH3 acts bi‐directionally to suppress slippage‐mediated insertions and deletions. These findings also reinforce the utility of tetranucleotide STR profiling as a sensitive readout of mismatch repair dysfunction and as a tool for characterizing genome stability in CRISPR‐edited cellular systems. In the penta‐ and hexanucleotide STR category (Figure S1C) the majority of loci exhibited minimal variation in repeat length across MSH3‐edited SW620 clones (E21, E22, and E23) when compared with the WT control. Most changes fell within a ±0.5 repeat window, suggesting a generally more stable behavior of long‐motif STRs relative to shorter motifs such as tri‐ and tetranucleotides. However, several loci demonstrated notable and reproducible alterations in STR length, indicating that specific sites remain sensitive to MMR perturbation. Among the expanded loci, the region proximal to *CNTN5* (chr11), characterized by an ATTTT motif, showed a marked increase from 7.5 repeats in WT to 9.0 in all three knock‐in clones, reflecting a +1.5 repeat expansion. This change, consistent across all mutant lines, suggests a site particularly vulnerable to insertion slippage in the absence of fully functional MSH3. Similar expansions were observed at the *CPEB1* locus (chr15, AAAG motif), where repeat counts rose from 7.0 in WT to 8.0 in the knock‐in clones (+1.0), and near *CBSL* (chr21, AAAC motif), where the repeat count increased from 5.5 to 7.0 (+1.5). These loci highlight focal points of microsatellite slippage within longer repeat motifs. Conversely, several loci demonstrated repeat contractions. For example, STRs near *SGO1* (chr3, AAAAC motif) contracted from 7.0 in WT to 6.5 in all edited clones (−0.5), while *FER* (chr5, CTTTT motif) and *ADAMTS9-AS2* (chr3, AAAAC motif) also showed reduced repeat counts in the knock‐in clones, suggesting preferential deletion‐prone instability at these sites following MSH3 disruption. Importantly, multiple loci—including *DYNC1H1*, *SNAP23*, *PCCA*, and *FAM53A*—exhibited stable repeat lengths across all genotypes. These sites may represent inherently stable STR regions or loci less dependent on MSH3 for slippage correction. Overall, these results indicate that while MSH3 inactivation can influence penta‐ and hexanucleotide STR stability, the effects appear more selective and less widespread compared to tetranucleotide motifs. The observed expansions and contractions at a subset of loci reinforce the motif‐specific and locus‐dependent role of MSH3 in maintaining microsatellite integrity. This adds depth to the understanding of how STR motif length and sequence context contribute to differential MMR vulnerability. Collectively, these findings reveal that CRISPR‐Cas9–mediated editing of the *MSH3* gene in SW620 colorectal cancer cells leads to motif‐specific microsatellite slippage, underscoring the critical role of MSH3 in maintaining repeat sequence integrity. Trinucleotide STRs exhibited a mixed profile, with both expansions and contractions observed, highlighting a balanced role for MSH3 in correcting insertion and deletion slippage events within coding or regulatory contexts. In contrast, tetranucleotide repeats were more prone to significant expansions, particularly at intergenic or intronic AT‐rich loci, suggesting that these motifs are especially vulnerable to insertional slippage in the absence of MSH3 surveillance. Meanwhile, longer motifs such as penta‐ and hexanucleotide STRs were generally stable, with only select loci exhibiting mild instability—indicating a more selective, locus‐dependent reliance on MSH3 for maintenance.

## 4. Discussion

Our previously reported six novel variants from Exons 8, 9, 20, 21, 22, and 23 of *MSH3* identified exclusively among AA‐CRC samples were likely pathogenic as determined by several bioinformatic approaches [25]. Here, we evaluated the biological significance of three of the discovered *MSH3* variants using various in vitro approaches. Our biological assays revealed no significant changes in cell morphology, proliferation (long‐ and short‐term), MSI/EMAST phenotype, subcellular localization of MSH3 protein, and MSH3‐MSH2 interaction between the variants (MSH3 Exons 21, 22 and 23) compared with WT MSH3 SW620 cells. Interestingly, MSI, a hallmark of MMR deficiency, plays a central role in the genomic evolution of multiple cancer types, particularly colorectal cancer. While traditional MSI assays have focused on mono‐ and dinucleotide repeats, emerging evidence underscores the significance of longer motifs—including tri‐, tetra‐, penta‐, and hexanucleotide STRs—in representing noncanonical instability events such as Elevated Microsatellite Alterations at Selected Tetranucleotide Repeats (EMAST) [50]. In this study, we leveraged CRISPR‐Cas9 genome editing to selectively disrupt *MSH3* in SW620 colorectal cancer cells and systematically profiled STR dynamics across clones using a panel of motif‐diverse loci. Our findings illuminate the motif‐ and locus‐specific impact of *MSH3* loss on microsatellite fidelity. Notably, tetranucleotide repeats emerged as the most instability‐prone class, with several loci showing consistent and reproducible expansions across all three edited clones (E21, E22, and E23). Interestingly, Exon 23 encodes the distal portion of the MutS_V clamp domain (aa 1044–1101), a region critical for MSH2–MSH3 heterodimer stability and clamp closure over DNA and Exon 23 (p.E1081K) potentially introduces a charge reversal at the MSH2–MSH3 interface. Our edited E23 clone showed reproducible tetranucleotide repeat expansions, with a stronger EMAST bias than E21 or E22, while leaving many mono/di repeats stable. These data indicate Exon 23 variants preferentially impair loop stabilization at longer repeats, explaining their distinct contribution to EMAST. STRs in the intergenic region near *LINC00550* (chr13, ATTT motif), as well as those adjacent to *FBXL7*, *MARCHF11*, and within the 3 ^′^ UTR of *DUSP28*, exhibited prominent repeat gains (+1.5 to +2 repeats). These expansions were strongly enriched in AT‐rich motifs (e.g., ATTT, AGAT, and GTTT), which are known to favor loop formation and slippage during DNA replication. In parallel, we also observed significant repeat contractions in loci such as *GPC5*, *ABCA13*, and *TMEM232*, suggesting that *MSH3* functions not only in resolving insertional mismatches but also prevents deletional slippage. These bidirectional instabilities are consistent with *MSH3*’s role as a critical partner in the MutS*β* complex (MSH2–MSH3), which specializes in recognizing loop mismatches typically formed at longer repeat sequences (Figure S1A–C). In contrast, penta‐ and hexanucleotide STRs displayed relatively stable profiles, with most loci showing changes ≤ 0.5 repeats. However, specific loci, such as those near *CNTN5*, *CPEB1*, and *CBSL*, still demonstrated measurable expansions, reinforcing the concept that MSH3′s influence on STR stability is motif‐ and context‐dependent. Interestingly, a subset of loci remained unchanged across WT and mutant backgrounds, such as *DYNC1H1* and *FAM53A*, suggesting that certain STRs are intrinsically stable or are regulated by alternative repair mechanisms not reliant on MSH3. Trinucleotide STRs showed an intermediate behavior, with some loci such as *GTF3C3* and *AKAP12* showing contractions, whereas others like *SLC25A12* exhibited expansions. These results suggest that *MSH3* contributes to stabilizing triplet repeats, a motif class frequently implicated in neurological and developmental disorders. Our data support a model in which *MSH3* safeguards STRs across various motif lengths, with a more profound effect observed in tetranucleotide contexts where loop mismatch substrates are optimal for MutS*β* recognition. From a biological standpoint, these results provide functional evidence linking *MSH3* disruption to the EMAST phenotype, which have been associated with advanced‐stage tumors, inflammation, and poor prognosis in CRC [50]. The observation of both expansions and contractions at STR loci reflects a loss of genomic surveillance and microsatellite type of sequence, length and context regulates frameshift mutation rates DNA MMR pathway which may lead to transcriptomic dysregulation or altered chromatin landscapes [51]. Moreover, this study highlights the utility of STR profiling as a high‐resolution molecular tool for monitoring MMR status beyond classical MSI markers. The ability to discriminate STR motif‐specific responses to *MSH3* loss adds a valuable dimension to functional genomics and cancer diagnostics. In summary, our findings demonstrate that *MSH3* plays a central role in maintaining STR integrity, particularly at tetranucleotide loci, and that its loss results in a measurable pattern of repeat expansions and contractions. This not only expands the spectrum of recognized MSI‐associated features in colorectal cancer but also provides a platform to explore STR‐based biomarkers of genomic instability, treatment response, or disease progression.

**Nomenclature**
MMR: DNA mismatch repair gene
MutS*α*
: MSH2‐MSH6 heterodimer
MutS*β*
: MSH2‐MSH3 heterodimer
*MSH3*
: MutS Homolog 3
MSH2: MutS homolog 2
CRISPR‐Cas9: Clustered regularly interspaced palindromic repeats‐CRISPR‐associated proteins
PERL: Post‐assembly analysis helper scripts
CRC: Colorectal cancer
AA: African Americans.
HTS: High‐throughput sequencing
PDB: Protein Data Bank
DAVID: Database for Annotation, Visualization, and Integrated Discovery
WGS: Whole genome sequencing
AI: Artificial Intelligence
MSI: Microsatellite Instability
EMAST: Elevated Microsatellite Alterations at selected tetranucleotide repeat
SIFT: Sorting Intolerant From Tolerant
PolyPhen‐2: Polymorphism Phenotyping v2
CADD: Combined Annotation Dependent Depletion
FATHMM MKL: Functional Analysis through Hidden Markov Models
DANN: Discriminative Analysis for Nonsynonymous Mutation
REVEL: Rapid Evolutionary Verification of Mutations Explorer
FATHMM: Functional Analysis Through Hidden Markov Models

## Author Contributions

Conceptualization: H.A. Methodology: T.Q., C‐C.R., M.R., K.C., S.A., S.T., J.A.S., M.K., and H.A. Investigation: H.B., M.R., and H.A. Visualization: H.B. and H.A. Funding acquisition: H.B., J.M.C., and H.A. Project administration: H.B. and H.A. Supervision: H.B. and H.A. Writing—original draft: M.R., H.B., and H.A. Writing—review/edits: M.R., H.B., J.M.C., S.T., and H.A.

## Funding

This study was supported by National Institutes of Health (10.13039/100000002; R01 CA258519).

## Conflicts of Interest

The authors declare no conflicts of interest.

## Supporting Information

Additional supporting information can be found online in the Supporting Information section.

## Supporting information


**Supporting Information 1** Figure S1A: Hierarchical clustering heat maps of STR repeat dynamics in *SW620 MSH3* mutant clones compared with wild type. The hierarchical clustering groups STR loci based on patterns of repeat count variation, highlighting regions undergoing contraction or expansion depicts heat map for trinucleotide STR repeat counts, associated gene(s), chromosome, STR motif, and the minimum observed difference across four samples: *SW620_WT* (wild‐type) and three *MSH3*‐mutated clones (*SW620_E21*, *SW620_E22*, *SW620_E23*), with color intensity representing STR repeat count.

**Supporting Information 2** Figure S1B: Hierarchical clustering heat maps of STR repeat dynamics in *SW620 MSH3* mutant clones compared with wild type. The hierarchical clustering groups STR loci based on patterns of repeat count variation, highlighting regions undergoing contraction or expansion depicts heat map for tetranucleotide STR repeat counts, associated gene(s), chromosome, STR motif, and the minimum observed difference across four samples: *SW620_WT* (wild‐type), and three *MSH3*‐mutated clones (*SW620_E21*, *SW620_E22*, *SW620_E23*), with color intensity represents STR repeat count.

**Supporting Information 3** Figure S1C: Hierarchical clustering heat maps of STR repeat dynamics in *SW620 MSH3* mutant clones compared with wild type. The hierarchical clustering groups STR loci based on patterns of repeat count variation, highlighting regions undergoing contraction or expansion depicts heat map for penta/hexa‐nucleotide STR repeat counts, associated gene(s), chromosome, STR motif, and the minimum observed difference across four samples: *SW620_WT* (wild‐type), and three *MSH3*‐mutated clones (*SW620_E21*, *SW620_E22*, *SW620_E23*), with color intensity represents STR repeat count.

**Supporting Information 4** Table S1: List of specific primer sequences of MSH3‐specific exons.

**Supporting Information 5** Table S2: List of guide RNA (gRNA) and donor DNA (dDNA) sequence of MSH3 specific exons.

**Supporting Information 6** Table S3: MSI/EMAST Primers with attached fluorophore.

## Data Availability

Data collected for the study, including de‐identified data and participant data, will be made available to others at publication via a signed data access agreement and at the discretion of the investigators′ approval of the proposed use of such data.
