# Supplementary figures and images for: Novel African American Colorectal Cancer MSH3 Variants Associate With Major Genomic Instability
Source: Hum Mutat. 2026 Jun 4;2026:5588764. doi: 10.1155/humu/5588764 (PMC13238251; doi:10.1155/humu/5588764)

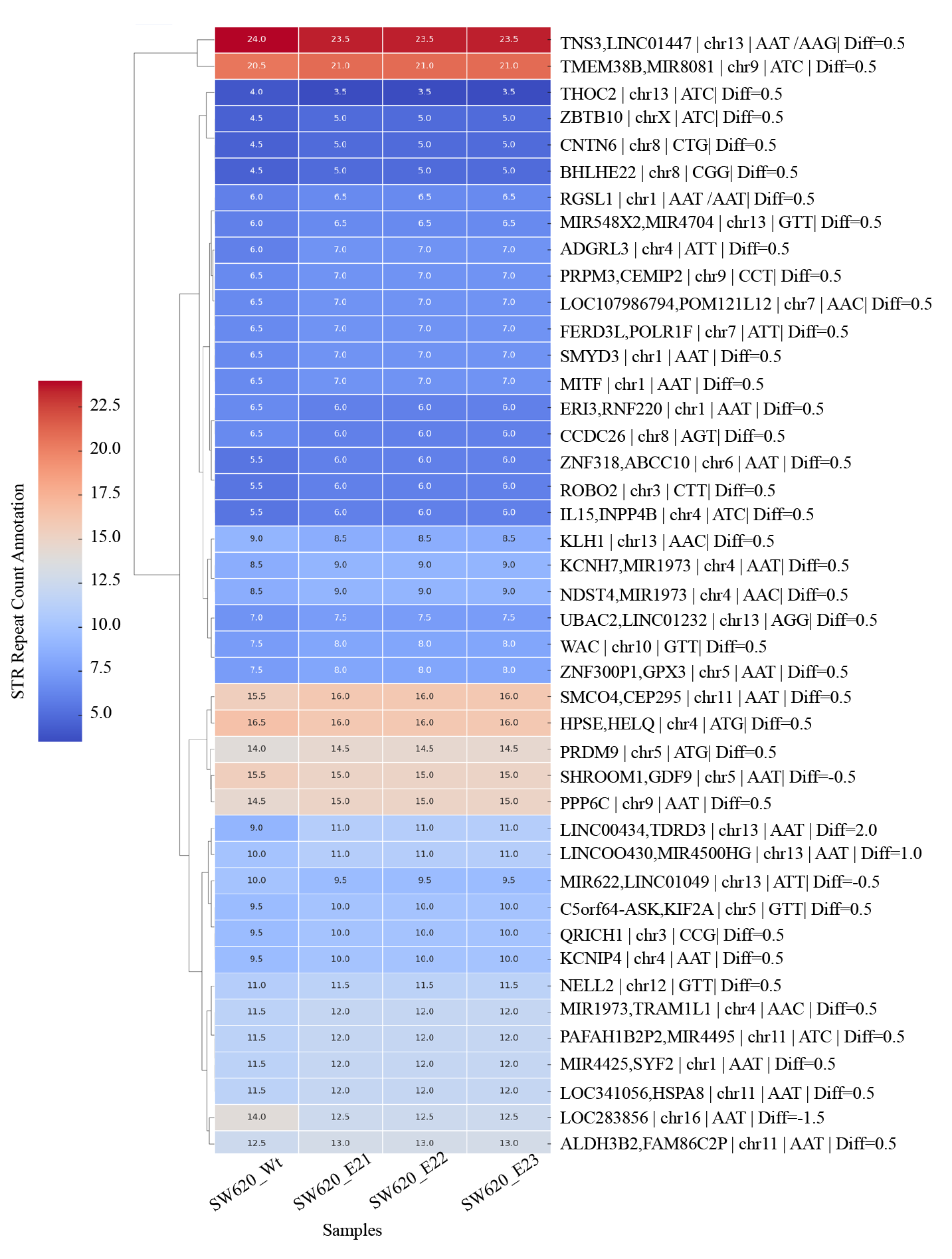

Supplement: Supplementary file 1 — Supporting Information 1 Figure S1A: Hierarchical clustering heat maps of STR repeat dynamics in SW620 MSH3 mutant clones compared with wild type. The hierarchical clustering groups STR loci based on patterns of repeat count variation, highlighting regions undergoing contraction or expansion depicts heat map for trinucleotide STR repeat counts, associated gene(s), chromosome, STR motif, and the minimum observed difference across four samples: SW620_WT (wild‐type) and three MSH3‐mutated clones (SW620_E21, SW620_E22, SW620_E23), with color intensity representing STR repeat count. [file HUMU-2026-5588764-s006.png]

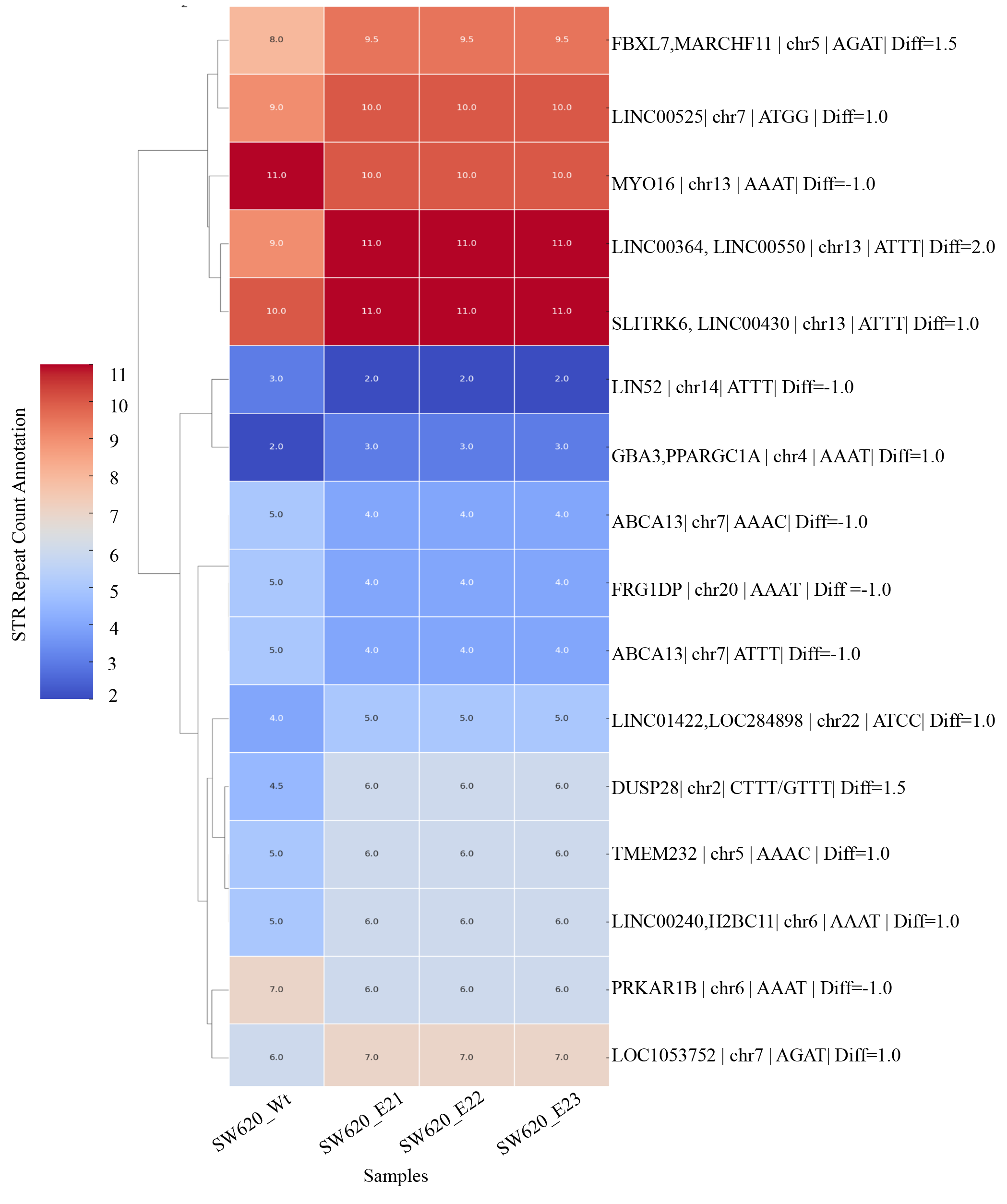

Supplement: Supplementary file 2 — Supporting Information 2 Figure S1B: Hierarchical clustering heat maps of STR repeat dynamics in SW620 MSH3 mutant clones compared with wild type. The hierarchical clustering groups STR loci based on patterns of repeat count variation, highlighting regions undergoing contraction or expansion depicts heat map for tetranucleotide STR repeat counts, associated gene(s), chromosome, STR motif, and the minimum observed difference across four samples: SW620_WT (wild‐type), and three MSH3‐mutated clones (SW620_E21, SW620_E22, SW620_E23), with color intensity represents STR repeat count. [file HUMU-2026-5588764-s001.png]

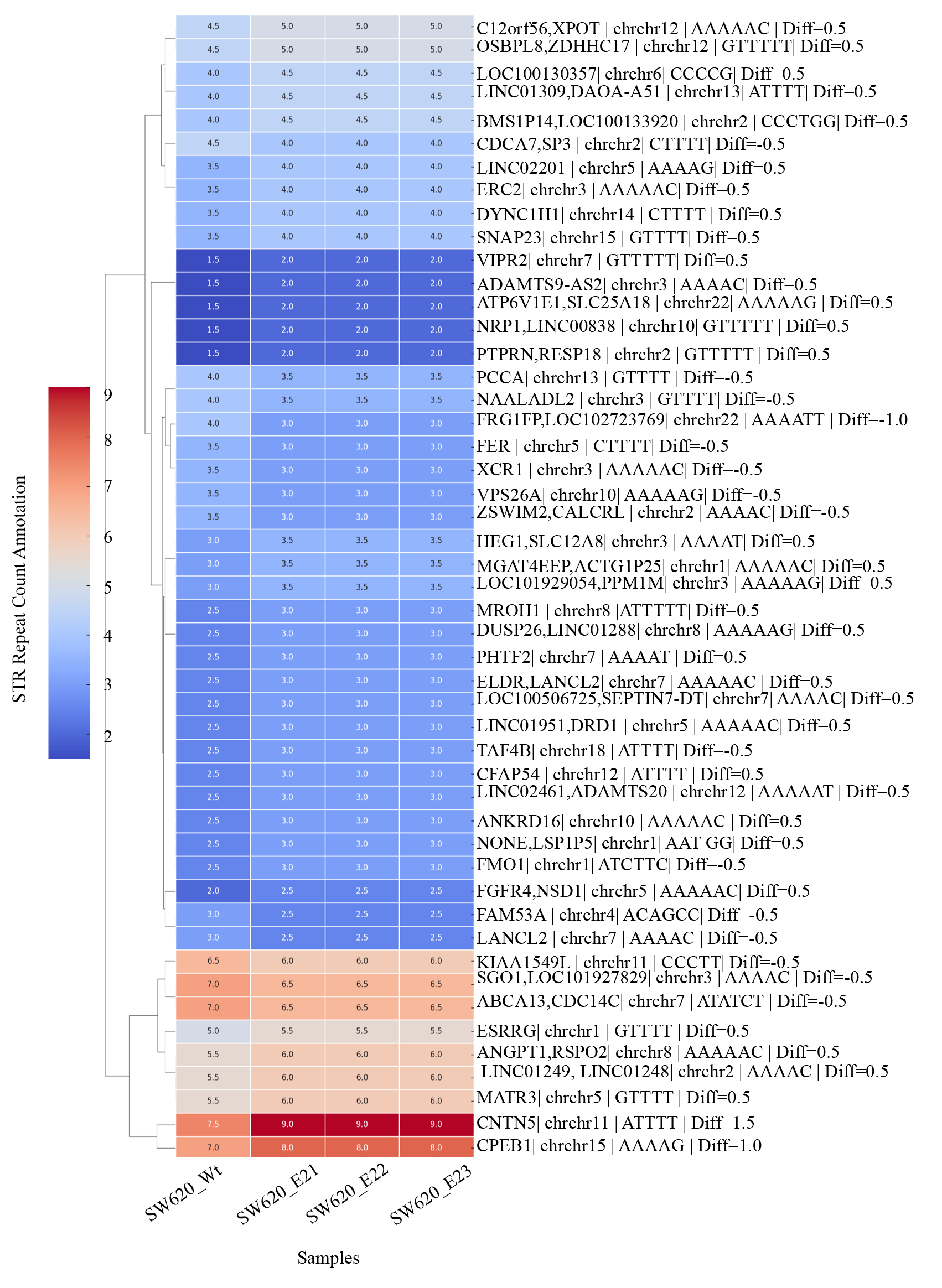

Supplement: Supplementary file 3 — Supporting Information 3 Figure S1C: Hierarchical clustering heat maps of STR repeat dynamics in SW620 MSH3 mutant clones compared with wild type. The hierarchical clustering groups STR loci based on patterns of repeat count variation, highlighting regions undergoing contraction or expansion depicts heat map for penta/hexa‐nucleotide STR repeat counts, associated gene(s), chromosome, STR motif, and the minimum observed difference across four samples: SW620_WT (wild‐type), and three MSH3‐mutated clones (SW620_E21, SW620_E22, SW620_E23), with color intensity represents STR repeat count. [file HUMU-2026-5588764-s002.png]
